# Effect of body mass index on mortality for diabetic patients with aortic stenosis

**DOI:** 10.18632/aging.206018

**Published:** 2024-07-24

**Authors:** Kai-Chun Chang, Li-Ting Ho, Kuan-Chih Huang, Jung-Chi Hsu, David Te-Wei Kuan, Ting-Tse Lin, Jen-Kuang Lee, Yen-Yun Yang, Shu-Lin Chuang, Lian-Yu Lin

**Affiliations:** 1 Division of Cardiology, Department of Internal Medicine, National Taiwan University Hospital, Taipei, Taiwan; 2 Department of Internal Medicine, National Taiwan University College of Medicine, Taipei, Taiwan; 3 Division of Cardiology, Department of Internal Medicine, National Taiwan University Hospital Hsin-Chu Branch, Hsinchu, Taiwan; 4 Department of Internal Medicine, National Taiwan University Hospital Jinshan Branch, New Taipei City, Taiwan; 5 Integrative Medical Database Center, Department of Medical Research, National Taiwan University Hospital, Taipei, Taiwan

**Keywords:** obesity, diabetes mellitus, aortic valvular stenosis

## Abstract

Background: Several studies suggest an “obesity paradox,” associating obesity with better cardiovascular outcomes in patients with type 2 diabetes mellitus (DM) or aortic stenosis (AS) compared to normal or underweight individuals. This study explores the impact of body mass index (BMI) on diabetic patients with AS.

Methods: Between 2014 and 2019, patients with DM who underwent echocardiography were analyzed. Outcomes included all-cause mortality, cardiovascular, and non-cardiovascular death. Patients were categorized as underweight, normal weight, or obese based on BMI (<18.5, 18.5 to 27, and >27 kg/m2, respectively).

Results: Among 74,835 DM patients, 734 had AS. Normal weight comprised 65.5% (n=481), underweight 4.1% (N=30), and 30.4% were obese. Over a 6-year follow-up, underweight patients had significantly higher all-cause mortality (HR 1.96, 95% CI 1.22 – 3.14, p = 0.005), while obese patients had significantly lower mortality (HR 0.79, 95% CI 0.68 - 0.91, p=0.001) compared to the normal group. Regarding etiologies, underweight patients had a higher risk of non-cardiovascular death (HR 2.47, 95% CI 1.44-4.25, p = 0.001), while obese patients had a lower risk of cardiovascular death (HR 0.66, 95% CI 0.50-0.86, p=0.003). Subgroup analysis showed a consistent trend without significant interaction.

Conclusions: BMI significantly impacts mortality in DM patients with AS. Being underweight is associated with worse non-cardiovascular death, while obesity is linked to improved cardiovascular death outcomes.

## INTRODUCTION

Aortic stenosis (AS) is a prevalent valvular heart disease, particularly affecting the elderly, with up to 12.4% prevalence in those aged 75 and older [1]. The advent of transcatheter aortic valve replacement (TAVR) has provided a treatment option with comparable long-term outcomes to surgical aortic valve replacement (SAVR) for severe AS [2–4]. Despite advancements in treatment, all-cause mortality in AS patients remains high, and nearly half experience non-cardiovascular death [5, 6]. Recent meta-analyses indicate that even moderate AS is linked to elevated mortality [7, 8], and diabetes mellitus contributes to AS progression and worse outcomes [9, 10].

While obesity is generally considered a risk factor for cardiovascular diseases and overall mortality [11], recent research has revealed a phenomenon termed the “obesity paradox,” indicating decreased cardiovascular mortality in patients with high cardiovascular risk, including those with type 2 diabetes mellitus (DM) and individuals undergoing TAVR [12–14]. The association in SAVR remains debated [13, 15, 16]. The impact of obesity on outcomes in high cardiovascular risk patients is still a subject of discussion. This study aims to investigate the influence of body mass index (BMI) on diabetic patients with AS.

## MATERIALS AND METHODS

### Study population and data collection

This is a retrospective hospital-based cohort study conducted at the National Taiwan University Hospital (NTUH), a tertiary medical center in Taiwan. To adhere to data privacy regulations, personal identities were encrypted, and all data were analyzed in a de-identified manner. The study protocol received approval from the institutional review board of the National Taiwan University Hospital Ethics Committee (Reference Number: 201701084MINC).

Patients aged over 50 years with a diagnosis of type 2 diabetes mellitus (DM) were consecutively screened from January 1, 2014, to December 31, 2019, using the National Taiwan University Hospital integrated Medical Database (NTUH-iMD). This database includes detailed medical information, such as diagnoses, laboratory data, imaging studies, and prescription records [17]. Among the DM cohort, 22,095 subjects (29.52%) underwent echocardiography, primarily for heart failure (HF), coronary artery disease (CAD), and pre-operative surveys.

Patients were enrolled if aortic stenosis was diagnosed through echocardiography, assessed according to established guidelines for hemodynamic measures [18]. Two echocardiography specialists independently reviewed the echocardiography of 776 patients in the cohort. The index date of the DM-AS cohort was the first AS diagnosis by echocardiography, and detailed data collection methods are described in previous studies [19, 20].

Briefly, the baseline characteristics including body mass index (BMI), hypertension (HTN), hyperlipidemia, coronary artery disease (CAD), acute coronary syndrome (ACS), myocardial infarction (MI), chronic obstructive pulmonary disease (COPD), peripheral arterial occlusive disease (PAOD) were obtained from the electronic health records (EHRs). Echocardiographic studies were performed with Phillips iE33 (Phillips, Bothell, WA, USA) and two-dimensional-guided M-mode measurements with a 3.0- or 3.5-MHz transducer. Left atrium (LA) size, left ventricle internal dimension in end-diastole (LVIDd) and end-systole (LVIDs), left ventricle ejection fraction (LVEF), tricuspid regurgitation peak gradient (TRPG), left ventricle mass (LVM), the averaged early (E’) diastolic peak velocities of tissue Doppler measurements at the lateral and septal sites, mitral inflow peak early (E), peak late (A) flow velocity, the E/E’ and E/A ratio were measured according to the recommendations of the American Society of Echocardiography [21].

### Outcome measurement

The study examined all-cause mortality, cardiovascular death, and non-cardiovascular death as outcomes. A central committee adjudicated death events, classifying them into cardiovascular and non-cardiovascular categories. The occurrence times of the outcomes of interest were determined using diagnosis codes from electronic health records. The index date for outcomes was defined as the date of diagnosis, and medical records were reviewed until the last clinical visit or death.

### Statistical analysis

The patients were categorized into three groups based on their BMI (BMI < 18.5 as underweight, 18.5≤BMI≤ 27 as normal, 27< BMI as obesity). Baseline demographics were presented as median values with the 25th to 75th interquartile range (IQR) due to non-normal distribution of continuous variables. Categorical variables were expressed as frequencies (percentage). Group comparisons utilized the ANOVA test or chi-squared test.

For outcome analyses, event-free survival curves were depicted using the Kaplan–Meier method, and differences in survival among groups were assessed with the log-rank analysis. Cox proportional hazard regression models provided hazard ratios (HRs) with 95% confidence intervals (CI) for endpoint risks. Univariable and multivariable linear regression analyses, using forward selection, assessed variables independently associated with endpoints.

The relationship between BMI as a continuous variable and the risk of all-cause mortality, cardiovascular (CV), and non-cardiovascular (non-CV) death was also explored using restricted cubic spline analyses without additional covariate adjustment. Knots were located at the 10th, 50th, and 95th percentiles of BMI. Statistical analysis was conducted using SAS software, version 9.4 (SAS Institute, Cary, NC, USA).

### Availability of data and materials

The data underlying this article will be shared on reasonable request to the corresponding author.

### Consent for publication

All authors consent this article for publication.

## RESULTS

### Baseline characteristics

From January 1, 2014, to December 31, 2019, 74,835 patients with diabetes mellitus underwent screening. Those without echocardiographically diagnosed aortic stenosis were excluded, as were those without documented BMI. A total of 734 patients with a concomitant diagnosis of diabetes mellitus and AS were included (see Supplementary Figure 1). The median age was 79 years, and 338 (43.6%) patients were male. The median baseline BMI was 24.8 kg/m2. Aortic stenosis severity comprised 25% severe disease and 75% mild to moderate disease. Patients were further categorized into three BMI groups (underweight: BMI < 18.5, normal: 18.5≤BMI≤ 27, obesity: 27< BMI). Clinical and demographic characteristics are detailed in Table 1 and Supplementary Table 1. There were 30 (3.8%) patients in the underweight group and 223 (28.7%) in the obesity group. Baseline clinical characteristics were comparable except for age and comorbidities with hypertension. The underweight group was significantly older, and the obesity group was younger with more patients having hypertension. Echocardiographic features showed a larger left atrium and ventricle size and left ventricle mass in the obesity group. The severity of AS was comparable between the BMI groups. As for medications, there are significantly more patients taking anti-hypertensive agents, including calcium channel blockers (CCB), angiotensin-converting enzyme inhibitors (ACEi), and angiotensin receptor blockers (ARB) in the obesity group. As for diabetes control, the ratio of patients requiring insulin injection were similar between BMI groups (40.0% vs. 42.2% vs. 45.7 %). There are more patients taking oral anti-diabetic agents including metformin, dipeptidyl peptidase IV inhibitor (DPP4 inhibitor) and sodium-glucose co-transporter 2 inhibitor (SGLT2i) in the obesity group.

**Table 1:** Baseline characteristic according to BMI.

	**BMI < 18.5 (N=30)**	**18.5 ≤ BMI ≤ 27 (N=481)**	**BMI ≥ 27 (N=223)**	**P-value**
Age, years	82.1 (8.1)	78.5 (9.6)	75.2 (9.6)	< 0.001
Male	9 (30.0)	218 (45.3)	100 (44.8)	0.26
BMI, kg/m2	17.1 (16.2-18.1)	23.7 (21.6-25.2)	29.6 (28.0-31.2)	< 0.001
Comorbidities				
Hypertension	18 (60.0)	279 (58.0)	155 (69.5)	0.014
Hyperlipidemia	9 (30.0)	214 (44.5)	105 (47.1)	0.208
Hyperuricemia	2 (6.7)	30 (6.2)	23 (10.3)	0.159
Heart failure	5 (16.7)	41 (8.5)	27 (12.1)	0.152
Atrial fibrillation	9 (30.0)	98 (20.4)	34 (15.3)	0.085
Stroke	3 (10.0)	41 (8.5)	11 (4.9)	0.210
CAD	6 (20.0)	171 (35.6)	81 (36.3)	0.203
Myocardial infarction	1 (3.3)	13 (2.7)	5 (2.2)	0.906
CABG	1 (3.3)	4 (0.8)	2 (0.9)	0.390
Chronic kidney disease	6 (20.0)	100 (20.8)	49 (22.0)	0.927
ESRD under hemodialysis	0 (0.0)	34 (7.0)	13 (5.8)	0.282
COPD	3 (10.0)	46 (9.6)	26 (11.7)	0.694
PAD	7 (23.3)	61 (12.7)	23 (10.3)	0.121
Lab data				
HbA1C, %	6.2 (5.6- 6.4)	6.6 (6.1-7.4)	6.8 (6.1-7.6)	0.049
Triglyceride, mg/dL	113.5 (85.0-135.5)	117.0 (86-159)	133.5 (98-179)	0.103
Total Cholesterol, mg/dL	143.5 (120-158)	154 (130-184)	158.5 (133-181)	0.245
LDL-C, mg/dL	70.5 (62-82)	88 (68-107)	89 (69-114)	0.040
HDL-C, mg/dL	38.5 (30-47)	41 (34-48)	41 (34-51)	0.547
AST, U/L	23.5 (16-35)	22 (18-30)	20.5 (16.5-26)	0.542
hsCRP, mg/dL	2.1 (1.2-3.7)	3.1 (1.1-7.8)	2.2 (0.7-6.3)	0.217
HCT, %	30.1 (26.9-33.4)	32.7 (28.0-37.4)	34.5 (29.3-39.8)	0.004
Echocardiography				
LA size, cm	4.0 (3.4-4.5)	4.1 (3.7-4.5)	4.3 (3.9-4.8)	0.002
LVIDd, cm	2.8 (2.4-3.3)	2.8 (2.5-3.3)	3.0 (2.6-3.4)	0.071
LVIDs, cm	4.6 (4.1-4.8)	4.7 (4.3-5.0)	4.9 (4.5-5.2)	< 0.001
LV mass, g	177.3 (150.5-206.5)	207.0 (170.3-246.6)	227.4 (191.9-274.9)	< 0.001
LVEF, %	63.7 (54.4-72.8)	68.1 (60.0-73.7)	69.0 (61.8-74.3)	0.355
E/A	0.8 (0.7-1.0)	0.8 (0.6-1.0)	0.8 (0.6-1.0)	0.804
E/e’	13.2 (10.4-22.7)	15.5 (12.2-21.2)	15.6 (11.7-18.4)	0.712
TRPG, mmHg	305.1 (240.0-342.5)	275.2 (246.5-315.1)	273.7 (242.4-310.3)	0.184
AS severity				
mild/ moderate	23 (76.7)	348 (72.4)	180 (80.7)	0.057

Data are expressed as median (interquartile range) or number (percentage). AS, aortic stenosis; AST, aspartate aminotransferase; BMI, body mass index; CAD, coronary artery disease; COPD, chronic obstructive pulmonary disease; ESRD, end stage renal disease; HCT, hematocrit; hsCRP, high sensitivity C-reactive protein; LA, left atrium; LDL-C, low density lipoprotein-cholesterol; LVEF, left ventricle ejection fraction; LVIDd, left ventricle internal diameter in diastole; LVIDs, left ventricle internal diameter in systole; LV, left ventricle; PAD, peripheral artery disease; TRPG, tricuspid regurgitation pressure gradient.

### Outcomes

**Figure 1:**
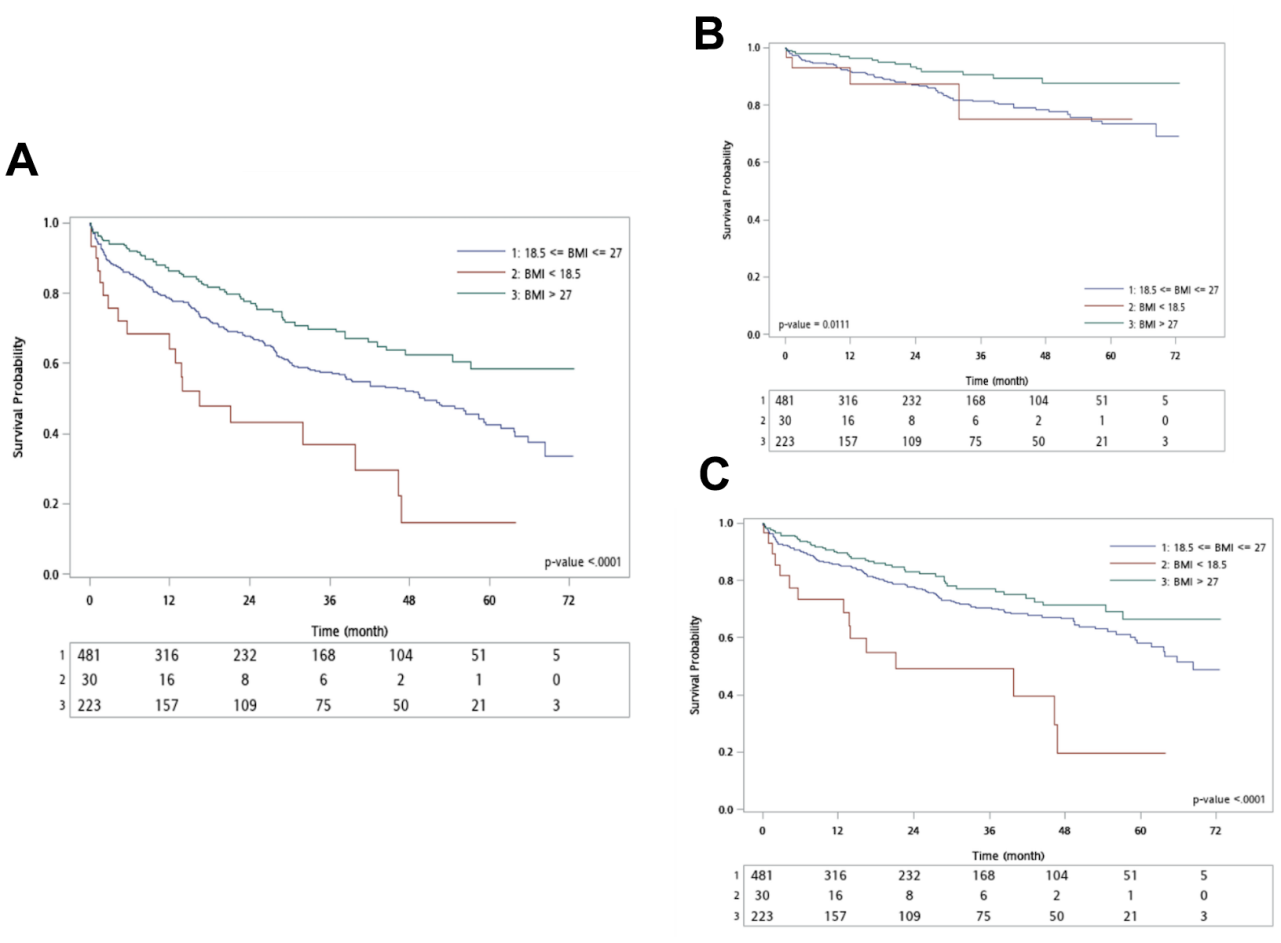
**Kaplan–Meier analysis for different outcomes**. (**A**) All-cause death; (**B**) Cardiac death; (**C**) Non-cardiac death.

With a median follow-up time of 34 months (IQR 13-54), 285 patients (38.8%) experienced outcomes of death, comprising 93 patients (32.7%) with cardiovascular death and 192 patients (67.3%) with non-cardiovascular death. The Kaplan-Meier curve and log-rank test revealed the highest all-cause mortality in underweight patients and the lowest in obesity patients (Figure 1A). Further classifying causes of death into cardiovascular and non-cardiovascular deaths, the underweight group showed significantly worse outcomes in non-cardiovascular death, while the obesity group exhibited significantly fewer cardiovascular deaths than the other two groups (Figure 1B, 1C).

In univariable analysis, underweight status and age ≥ 80 were associated with a significantly increased risk of all-cause mortality (see Supplementary Table 2). For underweight patients, the hazard ratio for non-cardiovascular death was significantly higher compared to the normal BMI group (HR: 2.59, 95% CI: 1.52-4.42; P-value: < 0.001), but not for cardiovascular death. Conversely, the obesity group was associated with a significantly lower all-cause mortality (HR: 0.62, 95% CI: 0.47-0.83; P-value: 0.001), with lower hazard ratios for both cardiovascular and non-cardiovascular deaths compared to the normal BMI group (Table 2). In patients aged ≥ 80, hazard ratios for both cardiovascular and non-cardiovascular death were significantly higher than in patients aged < 80 (see Supplementary Table 2).

**Table 2:** Hazard ratio for outcomes in different BMI groups.

	**All-cause mortality**		**Cardiovascular death**		**Non-cardiovascular death**	
	**HR (95% CI)**	**P-value**	**HR (95% CI)**	**P-value**	**HR (95% CI)**	**P-value**
Model 1: crude result						
18.5 ≤ BMI ≤ 27	Reference		Reference		Reference	
BMI < 18.5	2.11 (1.32-3.37)	0.002	1.24 (0.45-3.39)	0.678	2.59 (1.52-4.42)	< 0.001
BMI > 27	0.62 (0.47-0.83)	0.001	0.46 (0.27-0.79)	0.005	0.71 (0.51-1.00)	0.048
Model 2*						
18.5 ≤ BMI ≤ 27	Reference		Reference		Reference	
BMI < 18.5	1.96 (1.22-3.14)	0.005	1.07 (0.39-2.93)	0.900	2.47 (1.44-4.25)	0.001
BMI > 27	0.79 (0.68-0.91)	0.001	0.66 (0.50-0.86)	0.003	0.85 (0.07-1.01)	0.072

*Model adjusted for age, sex, hypertension, ESRD, severity of AS.

AS, aortic stenosis; BMI, body mass index; CAD, coronary artery disease; CI, confidence interval; ESRD, end stage renal disease; HR, hazard ratio.

In a multivariable Cox regression model considering age, sex, hypertension, end-stage renal disease (ESRD), severity of AS, and different BMI groups, underweight patients had a significantly higher all-cause mortality (adjusted hazard ratio (aHR): 1.96, 95% CI: 1.22 - 3.14, p=0.005) and a significantly higher hazard ratio for non-cardiovascular death (aHR: 2.47, 95% CI: 1.44-4.25, p= 0.001), but not for cardiovascular death (aHR: 1.07, 95% CI: 0.39-2.93; P=0.900). Conversely, the obesity group had a significantly lower all-cause mortality (aHR: 0.79, 95% CI: 0.68-0.91, p=0.001), significantly lower cardiovascular death (aHR: 0.66, 95% CI: 0.50-0.86, p=0.003), but no significant difference in non-cardiovascular death (aHR: 0.85, 95% CI: 0.07-1.01, p=0.072) compared to the BMI between 18.5-27 group (Table 2).

**Figure 2:**
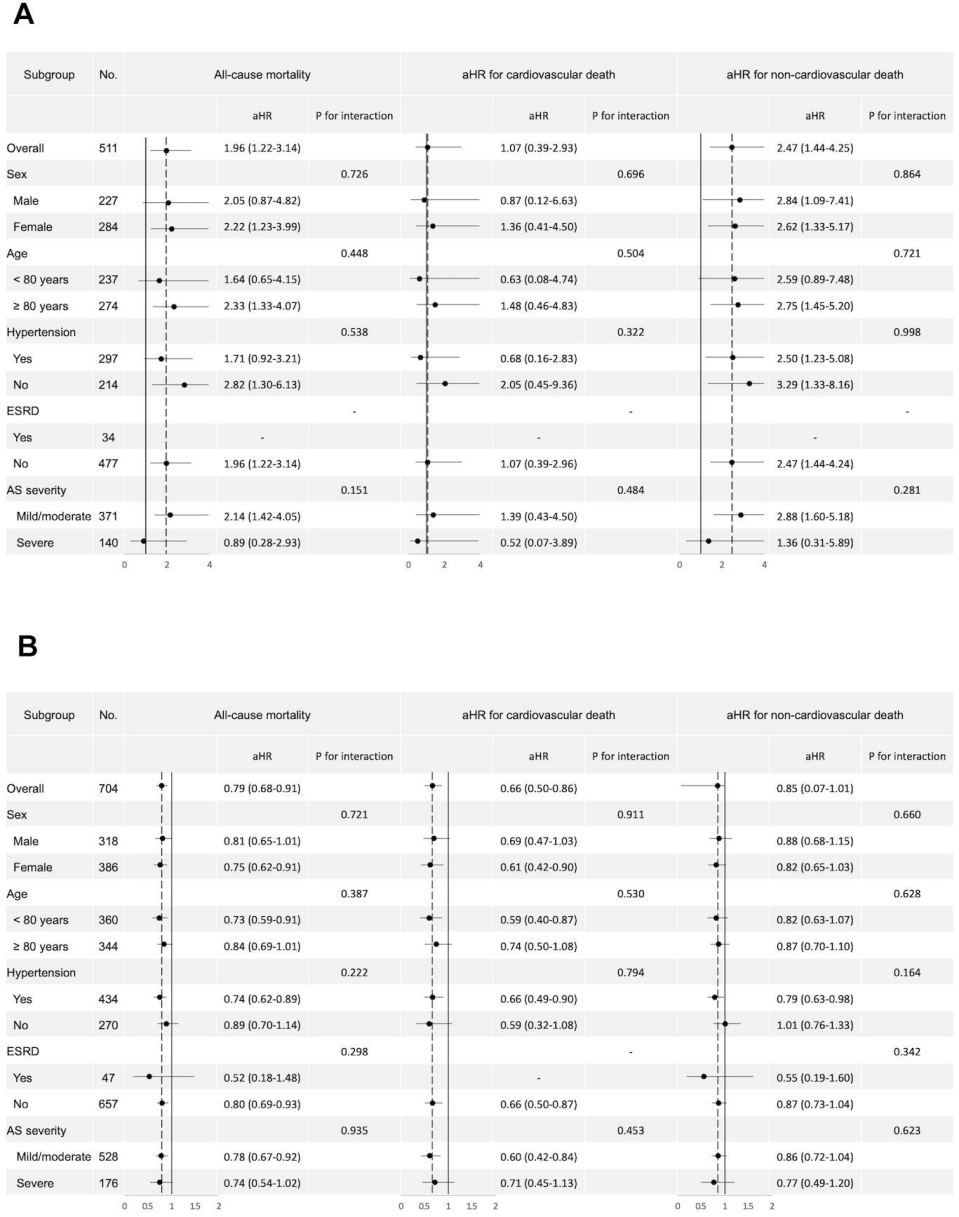
**Forest plot of subgroup analysis.** Forest plot of subgroup analysis for all-cause mortality, cardiovascular death, and non-cardiovascular death. (**A**) Subgroup analysis comparing underweight and normal BMI patients. (**B**) Subgroup analysis comparing obese and normal BMI patients. The plots display the adjusted hazard ratios (aHR) and 95% confidence intervals (CI) for various subgroups within the study population, including the p-values for interaction. BMI, body mass index; AS, aortic stenosis; ESRD, end stage renal disease.

Subgroup analysis, considering different baseline characteristics, revealed a consistent trend of increased all-cause mortality and non-cardiovascular death in underweight patients compared to normal-weighted patients. This trend was observed across subgroups based on sex, age, presence of hypertension, end-stage renal disease, or AS severity. Conversely, the lower risk for cardiovascular disease in obese patients compared to normal BMI patients was consistent across all subgroups (Supplementary Table 3 and Figure 2A, 2B). The interaction between BMI and subgroups was all insignificant.

**Figure 3:**
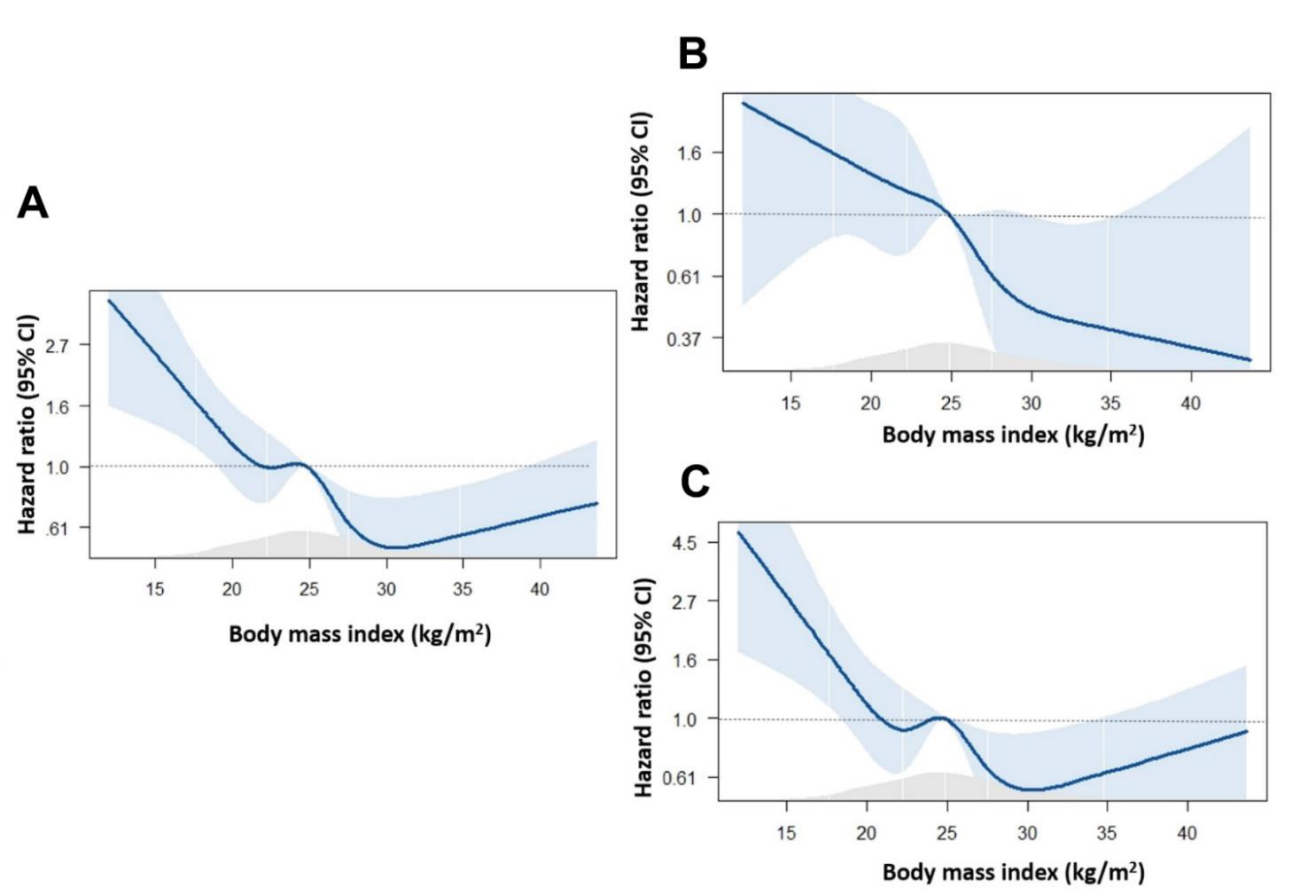
**Association between BMI and the risk of occurrence of events.** (**A**) All-cause death; (**B**) Cardiovascular death; (**C**) Non-cardiovascular death.

To further elucidate the interaction of BMI as a continuous variable with outcomes, including all-cause mortality, cardiovascular death, and non-cardiovascular death, restricted cubic spline analysis was applied. In Figure 3, all-cause mortality decreased as BMI increased until BMI exceeded 30. Cardiovascular death showed a negative association with BMI across available BMI levels, and non-cardiovascular death exhibited a U curve, with the lowest hazard ratio occurring when BMI was between 25 to 30.

## DISCUSSION

This longitudinal cohort study, focusing on diabetic aortic stenosis patients, revealed a 38.8% mortality rate over a median follow-up of 34 months. Non-cardiovascular death accounted for 67% of fatalities. Underweight individuals experienced notably worse all-cause mortality compared to those with a normal BMI, primarily driven by a significantly higher rate of non-cardiovascular deaths. Conversely, the obese group demonstrated significantly better all-cause mortality than the normal BMI group, largely due to a lower rate of cardiovascular deaths. Multivariable analysis, adjusting for key factors, confirmed these associations. Further analyses using restricted cubic splines indicated a negative association between BMI and cardiovascular death, with a steep increase in non-cardiovascular death when BMI fell below 25.

Our cohort exhibited a slightly higher proportion of non-cardiovascular deaths compared to previous studies involving aortic stenosis (AS) patients. Unlike prior research, our cohort specifically included AS patients with type 2 diabetes, a factor implicated in AS progression due to heightened proinflammatory processes, increased lipid accumulation, and accelerated calcification of valvular endothelial and interstitial cells [22]. The presence of diabetes and poor glycemic control also contributed to the increased incidence of non-cardiovascular causes of death, including infections [23]. In our cohort, with a median age of 79 years, notable comorbidities such as hypertension and stroke were observed. Aortic stenosis has been associated with a higher risk of ischemic stroke [24], which is the third leading cause of death in Taiwan and a significant contributor to complex disability. Stroke, in particular, is linked to a substantial risk of mortality, with up to 41% experiencing death within one year and a fivefold increase in mortality risk compared to the general population [25, 26]. The high prevalence of comorbidities, including diabetes mellitus, hypertension, and stroke, in our study may contribute to the elevated percentage of non-cardiovascular deaths [26].

The phenomenon of reduced mortality risk in obese patients, known as the “obesity paradox,” has consistently been observed in various high cardiovascular risk groups, including those with type 2 diabetes mellitus [12], heart failure, and coronary artery disease with revascularization. This paradox extends to patients with severe aortic stenosis undergoing medical treatment or transcatheter aortic valve replacement (TAVR) [14]. However, in patients receiving surgical intervention for severe aortic stenosis, the results have been controversial [13, 15, 16]. The pathophysiology behind the obesity paradox remains a subject of debate, with proposed explanations including lead time bias, differences in cardiopulmonary fitness, reverse causation, and variations in anthropometric indices [27, 28]. In our study involving a high cardiovascular risk cohort with diabetes mellitus and aortic stenosis, we observed the obesity paradox specifically in cardiovascular death. This finding aligns with several prior studies demonstrating a protective effect of obesity in patients undergoing intervention for severe aortic stenosis. Our study contributes additional insights into potential mechanisms underlying the obesity paradox in this specific population. Firstly, our cohort exhibited a high prevalence of cardiovascular co-morbidities such as coronary artery disease and heart failure, necessitating the use of guideline-directed medical therapies (GDMTs) like beta-blockers, angiotensin-converting enzyme inhibitors (ACEI)/angiotensin receptor blockers (ARB), or statins. It is noteworthy that these medications may be less well-tolerated in leaner patients [29, 30]. In our subgroup analysis, the protective effects of obesity were notably more pronounced in patients with milder diseases (age < 80 years and mild/moderate aortic stenosis), aligning with the observed tolerability of GDMTs in this patient subgroup. Secondly, the elevated prescription rate of GDMTs in the obese groups reflects a physician tendency to adopt a more aggressive treatment approach based on the belief of a higher cardiovascular risk in obese patients. The heightened disease awareness, close monitoring, and administration of cardioprotective drugs in the obese population may contribute to further modulating the outcomes. Third, in patients with severe AS, the outcome was highly associated with intervention, including TAVR and SAVR. Higher BMI was associated with a larger body surface area and possibly larger vessel size. Previous studies revealed that during TAVR, a higher sheath-to-femoral artery ratio (SFAR) was significantly associated with short-term mortality [31], which may explain the improved cardiovascular outcome in the obese AS patients.

Regarding non-cardiovascular death, our findings indicated a significantly higher risk associated with being underweight. A recent study on an Asian cohort undergoing transcatheter aortic valve replacement (TAVR) also reported a worse midterm prognosis in underweight patients [32]. This study revealed a significant association between being underweight and non-cardiovascular death, which accounted for up to 63% of deaths in underweight patients. This observation introduces the concept of a “lean paradox” in relation to non-cardiovascular death, emphasizing the potential impact of concomitant frailty and malnutrition in underweight individuals, particularly in the elderly population with aortic stenosis.

The study highlights the importance of considering BMI as a relevant factor in risk assessment and management strategies for diabetic patients with aortic stenosis. Clinicians should be vigilant about the heightened mortality risk associated with being underweight, emphasizing the need for comprehensive care addressing frailty and malnutrition in this subgroup. Conversely, the observed protective effect of obesity on cardiovascular death suggests potential benefits from aggressive treatment approaches and close monitoring in obese individuals, emphasizing the need for personalized and nuanced care strategies tailored to BMI categories in this patient population. Due to population aging, comprehensive geriatric assessment has gained increased emphasis recently. In addition to interventions for cardiovascular diseases, evaluating overall frailty, implementing aggressive cardiovascular risk reduction strategies, and promoting disease awareness have garnered growing interest for reducing both non-cardiovascular and cardiovascular deaths. Diabetes and aortic stenosis are highly prevalent in the geriatric population, and the increased mortality risk in this group necessitates thorough evaluation. Current screening tools for frailty, such as the FRAIL scale or the Clinical Frailty scale, require a detailed questionnaire for assessment. Our study sheds light on the possible mechanisms by which BMI affects clinical outcomes in this geriatric population and suggests that BMI may serve as a screening tool for frailty. Further studies are warranted to compare different measurements of frailty in this population.

### Limitation

The retrospective nature of our study limited the findings to associations between characteristics and outcomes, and the absence of access to symptoms for aortic stenosis was a constraint. Additionally, other obesity parameters such as waist circumference or percent body fat were not included in the analysis. In addressing these retrospective limitations, we conducted multivariable analysis involving outcome-associated factors and performed a subgroup analysis. To ensure the accuracy of severe aortic stenosis diagnosis, echocardiographic evaluations were independently reviewed by two specialists. While alternative obesity parameters are under research, BMI, being the most used in cardiovascular studies, was selected as the defining measure for obesity in our study.

## CONCLUSIONS

In conclusion, our study on diabetic patients with aortic stenosis underscored the significance of both cardiovascular and non-cardiovascular deaths as crucial contributors to overall mortality. The adverse association of BMI with both types of deaths was apparent in underweight patients. Notably, the protective effects of obesity were observed not only in the severe aortic stenosis group but also in milder cases. Proposed mechanisms include reduced frailty, enhanced tolerance to guideline-directed medical therapies (GDMTs), and heightened disease awareness in the obese group. Further studies are warranted to comprehensively elucidate these mechanisms and inform more targeted interventions.

## Supplementary Material

Supplementary Figure 1

Supplementary Tables
