# Supplementary material for: Effect of body mass index on mortality for diabetic patients with aortic stenosis
Source: Aging (Albany NY). 2024 Jul 24;16(14):11359–72. doi: 10.18632/aging.206018 (PMC11315379; doi:10.18632/aging.206018)
Supplement: Supplementary Figure 1 [file aging-16-206018-s001.pdf]

SUPPLEMENTARY FIGURE

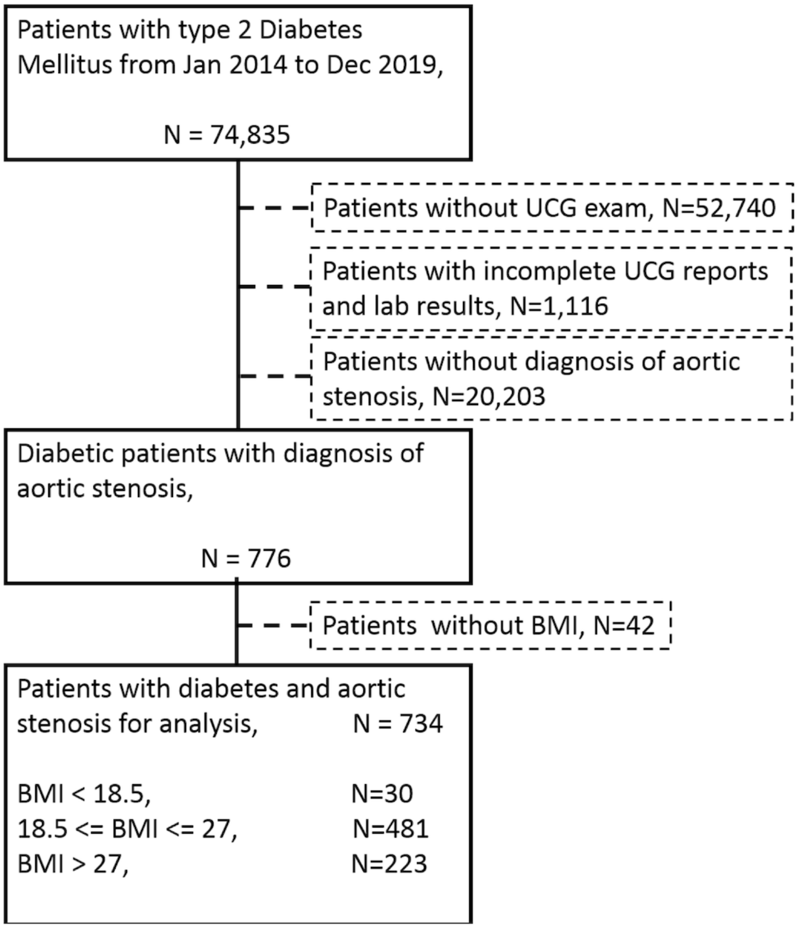

Supplementary Figure 1. Flow chart of patient selection. BMI, body mass index; UCG, ultrasound cardiogram.
