# Supplementary material for: Effect of body mass index on mortality for diabetic patients with aortic stenosis
Source: Aging (Albany NY). 2024 Jul 24;16(14):11359–72. doi: 10.18632/aging.206018 (PMC11315379; doi:10.18632/aging.206018)
Supplement: Supplementary Tables [file aging-16-206018-s002.pdf]

SUPPLEMENTARY TABLES

Supplementary Table 1. Baseline medications according to BMI.

	BMI < 18.5 (N=30)	18.5 ≤ BMI ≤ 27 (N=481)	BMI ≥ 27 (N=223)	P-value
CCB	18 (60.0)	284 (59.0)	156 (70.0)	0.020
BB	16 (53.3)	239 (49.7)	117 (52.5)	0.756
ACEi/ ARB	13 (43.3)	260 (54.1)	143 (64.1)	0.014
Diuretics	20 (66.7)	281 (58.4)	135 (60.5)	0.616
Statin	14 (46.7)	230 (47.8)	126 (56.5)	0.092
Insulin	12 (40.0)	203 (42.2)	102 (45.7)	0.636
Metformin	12 (40.0)	177 (36.8)	107 (48.0)	0.019
SGLT2i	0 (0.0)	24 (5.0)	20 (9.0)	0.043
DPP4i	10 (33.3)	216 (44.9)	118 (52.9)	0.045
Sulfonylurea	10 (33.3)	173 (36.0)	84 (37.7)	0.854
GLP1 agonist	0 (0.0)	1 (0.0)	4 (1.8)	0.053
Anti-coagulant	7 (23.3)	87 (18.1)	50 (22.4)	0.352
Antiplatelet agents	12 (40.0)	287 (59.7)	130 (58.3)	0.105

Data are expressed as number (percentage). ACEi, angiotensin converting enzyme inhibitor; ARB, angiotensin receptor blocker; BB, beta-blockers; CCB, calcium channel blocker; DPP4i, dipeptidyl peptidase IV inhibitor; GLP1 agonist, glucagon-like peptide 1 agonist; SGLT2i, sodium-glucose co-transporter 2 inhibitor.

Supplementary Table 2. Univariable cox regression analysis model for mortalities.

	All-cause mortality		Cardiovascular death		Non-cardiovascular death	
	HR (95% CI)	P-value	HR (95% CI)	P-value	HR (95% CI)	P-value
BMI group						
18.5 ≤ BMI ≤ 27	Reference		Reference		Reference	
BMI < 18.5	2.11 (1.32-3.37)	0.002	1.24 (0.45-3.39)	0.678	2.59 (1.52-4.42)	< 0.001
BMI > 27	0.62 (0.47-0.83)	0.001	0.46 (0.27-0.79)	0.005	0.71 (0.51-1.00)	0.048
Male vs. female	1.04 (0.83-1.32)	0.729	1.10 (0.73-1.66)	0.635	1.01 (0.76-1.35)	0.923
Age ≥ 80 vs. < 80	2.04 (1.71-2.59)	< 0.001	1.64 (1.09-2.48)	0.018	2.28 (1.70-3.06)	< 0.001
AS severity						
severe vs. mild/moderate	0.91 (0.70-1.19)	0.483	1.48 (0.96-2.26)	0.073	0.69 (0.48-0.98)	0.036
Hypertension (yes vs. no)	1.26 (0.99-1.60)	0.063	1.51 (0.98-2.33)	0.061	1.15 (0.86-1.54)	0.338
ESRD (yes vs. no)	0.70 (0.39-1.25)	0.227	0.35 (0.09-1.41)	0.139	0.88 (0.46-1.66)	0.689
CAD (yes vs. no)	1.01 (0.79-1.29)	0.933	1.46 (0.97-2.21)	0.073	0.83 (0.61-1.14)	0.249

AS, aortic stenosis; BMI, body mass index; CAD, coronary artery disease; CI, confidence interval; ESRD, end stage renal disease; HR, hazard ratio.

Supplementary Table 3. Subgroup analysis with multivariable cox regression model.

BMI < 18.5 vs. 18.5 ≤ BMI ≤ 27						
**Subgroup**	**All-cause mortality**		**Cardiovascular death**		**Non-cardiovascular death**	
	**HR (95% CI)**	**P-value**	**HR (95% CI)**	**P-value**	**HR (95% CI)**	**P-value**
Sex	P for interaction	0.726		0.696		0.864
Male (N=227)	2.05 (0.87-4.82)	0.102	0.87 (0.12-6.63)	0.896	2.84 (1.09-7.41)	0.033
Female (N=284)	2.22 (1.23-3.99)	0.008	1.36 (0.41-4.50)	0.609	2.62 (1.33-5.17)	0.005
Age	P for interaction	0.448		0.504		0.721
< 80 years (N=237)	1.64 (0.65-4.15)	0.295	0.63 (0.08-4.74)	0.655	2.59 (0.89-7.48)	0.079
≥ 80 years (N=274)	2.33 (1.33-4.07)	0.005	1.48 (0.46-4.83)	0.513	2.75 (1.45-5.20)	0.002
Hypertension	P for interaction	0.538		0.322		0.998
Yes (N=297)	1.71 (0.92-3.21)	0.092	0.68 (0.16-2.83)	0.593	2.50 (1.23-5.08)	0.011
No (N=214)	2.82 (1.30-6.13)	0.009	2.05 (0.45-9.36)	0.356	3.29 (1.33-8.16)	0.010
ESRD	P for interaction	-				-
Yes (N=34)	-	-	-	-	-	-
No (N-477)	1.96 (1.22-3.14)	0.006	1.07 (0.39-2.96)	0.890	2.47 (1.44-4.24)	0.001
AS severity	P for interaction	0.151		0.484		0.281
Mild/moderate (N=371)	2.14 (1.42-4.05)	0.001	1.39 (0.43-4.50)	0.583	2.88 (1.60-5.18)	<0.001
Severe (=140)	0.89 (0.28-2.93)	0.858	0.52 (0.07-3.89)	0.522	1.36 (0.31-5.89)	0.682
**BMI > 27 vs. 18.5 ≤ BMI ≤ 27**						
**Subgroup**	**All-cause mortality**		**Cardiovascular death**		**Non-cardiovascular death**	
	**HR (95% CI)**	**P-value**	**HR (95% CI)**	**P-value**	**HR (95% CI)**	**P-value**
Sex	P for interaction	0.721		0.911		0.660
Male (N=318)	0.81 (0.65-1.01)	0.063	0.69 (0.47-1.03)	0.067	0.88 (0.68-1.15)	0.360
Female (N=386)	0.75 (0.62-0.91)	0.004	0.61 (0.42-0.90)	0.013	0.82 (0.65-1.03)	0.087
Age	P for interaction	0.387		0.530		0.628
< 80 years(N=360)	0.73 (0.59-0.91)	0.005	0.59 (0.40-0.87)	0.008	0.82 (0.63-1.07)	0.143
≥ 80 years (N=344)	0.84 (0.69-1.01)	0.070	0.74 (0.50-1.08)	0.120	0.87 (0.70-1.10)	0.245
Hypertension	P for interaction	0.222		0.794		0.164
Yes (N=434)	0.74 (0.62-0.89)	0.001	0.66 (0.49-0.90)	0.009	0.79 (0.63-0.98)	0.033
No (N=270)	0.89 (0.70-1.14)	0.367	0.59 (0.32-1.08)	0.085	1.01 (0.76-1.33)	0.946
ESRD	P for interaction	0.298				0.342
Yes (N=47)	0.52 (0.18-1.48)	0.219	-	-	0.55 (0.19-1.60)	0.273
No (N-657)	0.80 (0.69-0.93)	0.003	0.66 (0.50-0.87)	0.003	0.87 (0.73-1.04)	0.120
AS severity	P for interaction	0.935		0.453		0.623
Mild/moderate (N=528)	0.78 (0.67-0.92)	0.004	0.60 (0.42-0.84)	0.003	0.86 (0.72-1.04)	0.133
Severe (N=176)	0.74 (0.54-1.02)	0.064	0.71 (0.45-1.13)	0.146	0.77 (0.49-1.20)	0.248

The hazard ratio was adjusted for outcome-associated factors including age, sex, hypertension, ESRD, severity of AS.
AS, aortic stenosis; BMI, body mass index; CAD, coronary artery disease; CI, confidence interval; ESRD, end stage renal disease; HR, hazard ratio.
